# Secondary Forests and Agrarian Transitions: Insights from Nepal and Peru

**DOI:** 10.1007/s10745-021-00224-1

**Published:** 2021-03-02

**Authors:** Adam Pain, Kristina Marquardt, Dil Khatri

**Affiliations:** 1 Department of Urban and Rural Development, Swedish University of Agricultural Science, Uppsala, Sweden; 2 Southasian Institute of Advanced Studies, Baneshwor, Kathmandu, Nepal

**Keywords:** Forest and agrarian transitions, Secondary forest, Territory, Social differentiation, Rural households, Indigenous peoples, Nepal, Peruvian Amazon

## Abstract

We provide an analytical contrast of the dynamics of secondary forest regeneration in Nepal and Peru framed by a set of common themes: land access, boundaries, territories, and rights, seemingly more secure in Nepal than Peru; processes of agrarian change and their consequences for forest-agriculture interactions and the role of secondary forest in the landscape, more marked in Peru, where San Martín is experiencing apparent agricultural intensification, than in Nepal; and finally processes of social differentiation that have consequences for different social groups, livelihood construction and their engagement with trees, common to both countries. These themes address the broader issue of the necessary conditions for secondary forest regeneration and the extent to which the rights and livelihood benefits of those actively managing it are secured.

## Introduction

Tropical forests have a core regulatory function in global climate systems, but even if they are conserved, existing primary or near climax tropical forests are unlikely to be sufficient to maintain this role (Chazdon 2014). Since secondary forests now provide at least 70% of tropical forest cover (FAO 2010) and make a critical contribution to the maintenance of global climate systems it is important to identify the conditions under which such forests are established and maintained. But secondary forests are too often regarded as degraded and too little attention is given to their diverse formations (Chazdon *et al.* 2016), the ecosystem services they provide, and crucially the contribution that smallholders can make in managing such forests for livelihood benefits (Hecht 2014). Their diversity reflects the nature of the disturbances that created them, the institutional regimes within which those are embedded, and the resulting forest regeneration processes. A more nuanced definition of secondary forest is required to capture the spectrum of secondary forest types in terms of their origins, scale, and use values to encompass swidden agriculture systems, managed forests, social forestry, and assisted regeneration of old growth forests (Pain *et al.* 2020).

Current thinking on secondary forest formation draws heavily on ideas of the normative models of agrarian and forest transitions that are linked to notions of stages of development (Mather 1992; Timmer 2014). But the conditions that might have generated such transitions in the past are no longer present. Equally the notion of separate categories of forest and agriculture have been questioned (Hecht 2014). Secondary forests (Pain *et al.* 2020), within and outside the forest boundary, lie in a transition zone for foresters and agronomists. Here we explore the conditions under which secondary forest regeneration takes place and whether these conditions necessarily guarantee the security of users of forest resources who have a vested interest in its management through a comparative analysis of the dynamics of smallholder actions in various types of secondary forest regeneration and the agrarian conditions associated with these. We draw from long term research in the mid-hills of Nepal (Marquardt *et al.* 2016) and in the San Martín region of the Peruvian Amazon (Marquardt *et al.* 2019).

Although Nepal and Peru are both experiencing processes of modernisation their settlement patterns differ markedly. The Nepalese hills are densely settled, the population depends on a remittance economy, and the margins of cultivation have long been fixed. Very little primary forest remains in the hills. In contrast the Peruvian Amazon is sparsely populated, remains a frontier region (Rasmussen and Lund 2018), and the forest boundary is not fixed. But from an analytical perspective the differences should not mask the fact that a common analytical framework can contextualise their forest settings and deepen understanding of forest and agrarian processes of change. We seek analytical generalisation (Lund 2014) drawing on and refining key concepts of context, forest governance, and agrarian change. Central to our analysis is the use of categories and normative models to characterize disparate practices on the ground.

In Nepal, democratic decentralisation of the forests and community forest development generated from a background of Himalayan degradation (Thompson *et al.* 1986) have been described by Paudel (2016) as a re-invention of the commons and a case of accumulation without dispossession. However, the terms and conditions under which this occurred raise questions as to who has benefited. There has been a significant recovery of secondary forest in the Nepalese mid- hills (Niraula *et al.* 2013; Birch *et al.* 2014), although the mosaic of forest cover and agroecological variability of mountain landscapes demands careful attention to micro-contexts in assessing the nature and species composition of that recovery and its benefits (Marquardt *et al.* 2016).

In contrast, the Amazonian region of Peru holds the fourth largest area of primary or old-growth tropical forest in the world (FAO 2010). But forest cover is being lost at an estimated rate of 123,000 ha per year (Hansen *et al.* 2013) to road development, agricultural intensification and an influx of migrants, although the Peruvian state sees smallholders’ swidden practices as the main culprit for deforestation in this region (MINAG 2002). In response, forest conservation programmes have been designed largely following a fortress forestry approach prioritising old growth forest with less attention given to secondary forests.

At first sight these country contrasts seem to offer insights into the drivers of reforestation and deforestation from a perspective of forest transition theory. Such analysis would focus on the changes in forest cover and quality but ignore the wider dynamics of forest and agrarian change and their contingent and conjunctural circumstances, including land use where an agricultural frontier is either fixed or open and the competition for land with shrinking farm sizes (and landlessness) or by expansionist capitalist agriculture. It would also not address the contrast between an agrarian economy that has largely failed to make a transition to capitalist agriculture and outmigration and remittances have become a key component of the rural economy (Sugden *et al.* 2017) and the situation of migrants attracted by land availability in the Peruvian Amazon leading to crop booms. Nepal has seen the integration of agricultural and forest land leading to a retreat of the cultivation margins and a re-treeing of the agrarian landscape outside the forest boundary areas. In the Peruvian Amazon, the increasing number of large oil palm plantations and smallholder commercial agriculture of tree crops and competition for land between large scale and small-scale farming are driving forest loss to which the creation of forest conservation zones has been an ineffectual response.

We first discuss the debates on agrarian and forest transitions before examining in more detail these processes in each country. We conclude with a discussion of the limits of existing land use categories and the redundancy of classic models of transitions and the implications for securing the regeneration of secondary forests.

### Understanding Processes of Agrarian and Forest Transitions

Modernisation orthodoxy (Timmer 2014) champions the dynamics of structural change as the normative route through which development should occur: the rise of capitalist agriculture leads to agricultural intensification, increasing farm productivity, the shedding of labour from agriculture, and the growth of a non-agricultural economy that absorbs agrarian labour. This premise also underlies the normative model of forest transition whereby agriculture retreats from marginal lands leading to forest recovery through secondary forest regeneration (Mather 1992; Rudel *et al.* 2010). It is also the orthodoxy that informs Peru’s approach to forest conservation, but in Nepal secondary forest regeneration has not been achieved through such a transition. Moreover, while historically this agrarian structural change may have been the Western experience and more recently that of certain East Asian states under specific state managed conditions (Timmer 2014), this is not what seems to be happening elsewhere. There is increasing evidence of agrarian transitions failing or becoming blocked, e.g., in India (Lerche *et al.* 2013) and Indonesia (Li 2014). This has resulted in increasing landlessness, migrant labour securing at best seasonal or part time employment in the informal sector of urban economies, and the rise of distributional rural economies (Ferguson 2015; Pain and Huot 2018). These households remain rurally based to secure informal support through personal social relationships but find few opportunities for remunerative work.

A market-based approach to agrarian transitions focuses almost exclusively on land and labour productivity and the role of technology in increasing these. Political economy approaches in contrast draw attention to the social relations that are central to production and productivity and that shape how farming is organised. An interest in the class based nature of agrarian change, processes of social differentiation, the nature of agrarian transitions, and the consequences of capitalist development for rural classes has long been central to agrarian political economy approaches (Akram-Lodhi and Kay 2010).

But the evidence points to the fracture of the interlinkages between capitalist agriculture and industrial development. For example, in India strong industrial growth has drawn capital from outside agriculture (Lerche *et al.* 2013) resulting in growth that does not create employment or pull labour out of agriculture (Li 2013). This suggests that the classic agrarian transition model is no longer necessary (Bernstein 2006) as the structural transformations of the past may no longer be possible. This raises questions about the premises of the classic forest transition model and expectations of increasing land use efficiency moving people out of agriculture, leading to land abandonment and secondary forest regeneration.

Agrarian political economy perspectives increasingly look to the consequences of expanding capitalist agriculture, uneven processes of capital accumulation, and forms of social differentiation, all of which are becoming more complex combining both on farm and forest elements creating variation and complexity in forest cover dynamics (Hecht 2014). There is also greater involvement of farming households in both rural and urban off farm labour markets. Much of this employment is in the informal sector with degrees of self-employment, petty production, and trading offering at best meagre remuneration. The persistence of classes of farmers whose primary aim is subsistence or simple reproduction (Bernstein *et al.* 2018) has encouraged a return to Chayanov’s analysis of the dynamics and logic of peasant household economies (van der Ploeg 2014), even if rural economies are now permeated by wider class relations inherent to capitalist production. The persistence of small farms that may contain secondary forest points to the uneven development of capitalism in farming.

Since normative models of agrarian and forest transitions do not fit with observed land use changes, examination of the contrasts between Peru and Nepal allows us to focus on the specific conjunctures that have generated the outcomes in each context, which have been shaped by economic, agroecological, geographic, agronomic, social, and institutional elements, and their dynamic interaction over time (Li 2014:16) to provide a selective account of distinctive context specific elements that may explain their specific forest outcomes and their wider relevance.

## Methods

We draw from long term research in Dolokha, Ramechhap, and Chitwan districts in the mid-hills of Nepal, and the lower slopes of the Andean range in the San Martín region of the Peruvian Amazon, which are two core sites in an ongoing research programme started in 2012 as a comparative enquiry into forest and agricultural land use in relation to ecosystem service management that evolved from 2015 into a broader investigation into the dynamics of landscape change, forest and agrarian transitions and the role of secondary forest in land use systems. We discuss details of context, research sites, forest types, and land use below (for Nepal: Marquardt *et al.* 2016; Khatri *et al.* 2018; Marquardt *et al.* 2020; for Peru Egerlid *et al.* 2016; Marquardt *et al.* 2019).

In both Peru and Nepal, forest is closely connected to agricultural livelihoods and the research sites were chosen to capture the diversity of the agroecological context, livelihoods, land use pressures, degree of commercialisation of farming and access to markets, and changes over a 20 year period. By focusing on smallholders we aim to capture a broad spectrum of forestry and agrarian transitions and landscape change dynamics in the sites. Common to both countries has been close collaboration with local research partners and the use of qualitative methods that have combined village studies, landscape mapping, and household and key informant interviews. Key areas of enquiry included household use of the forest, natural resource management activities in relation to water sources, soil fertility, use of non-timber forest products (NTPFs), forest composition and landscape management and how this has changed.

## Nepal

### Context

Until the nineteenth century, Nepal’s Hindu state had a strong political control over its hill populations (Shneiderman 2010) reflected in complex and extractive land rights and taxation systems that evolved over time (Regmi 1977). Grounded on a caste system that endures despite being abolished in 1963, social differentiation between high and low castes, caste and non-caste people, and distinctions between higher status hill people and lower status *terai* (plains) people still affect livelihood opportunities. These are reflected in income and consumption poverty outcomes with those at the bottom of the social hierarchy distinctly worse off. The World Bank (2007:20) wrote: ‘differences in consumption levels can be called the ‘penalty’ that certain groups pay because of their caste, ethnicity or religious identity.’ Most rural households do not have sufficient land to provide subsistence and according to Alden Wily *et al.* (2009) up to 58% of Nepalese farmers or 2.7 million rural households are functionally landless with less than 0.5 ha of farm land and depend on access to forest resources for survival.

Forests have histories on which their current institutional and social identities are built (Hecht *et al.* 2016). Although Nepali law has historically recognised forests as a form of state or communal property, forests became an expanding source of revenue (Regmi 1988) for the Nepali state during the nineteenth century with the export of Sal (*Shorea robusta*) timber for railway construction in India by the British colonial authorities. However, the allocation of private property rights through tax free grants to individuals connected to the ruling elite meant that significant areas of forests land, possibly up to a third of the total area both in the *terai* and hills were under private ownership. After 1951 with the emergence of a democratic movement, forests were nationalised in 1957 leading to major deforestation. They remained state property after the Private Forest Nationalization Act 1957 although the initiation of Panchayat forests in the 1970s brought a degree of decentralisation of forest management, promoted forest protection and plantation, and contributed to the emergence of local community participation in forest management and secondary forest regeneration.

Until the late 1980s the main role of forestry officers was revenue collection and conservation, but lack of effective conservation strategies led to a severe decline in forest cover (Metz 1991). Following the 1989 Forest Master Plan, the Forest Act of 1993, reflecting the move towards democracy after the 1991 elections, provided the framework for the decentralisation of forest management to forest user groups and the development of the Community Forest Programme (Acharya 2002). By 2018 this covered more than one fourth of the country’s forest area with over 22,250 Community Forest Groups (CFUGs) managing about 22.2 million ha of forests (DoF Hamro Ban 2018) and led to significant forest recovery, particularly in the mid-hills, through forest plantation and natural regeneration of tree cover (Yadav *et al.* 2003; Niraula *et al.* 2013) that improved the supply of forest products such as fodder and fuel wood as well as the volume of standing timber (Gautam *et al.* 2003; Adhikari *et al.* 2007; Birch *et al.* 2014).

### Governing the Forest

Paudel’s (2016) characterisation of the expansion of community forestry in Nepal as a process of accumulation without dispossession was based on the observation that CFUG development has engaged communities that provide the labour to manage the forests but on terms and conditions set externally. CFUG governance has increasingly been driven by neo-liberal market forces, i.e., management for carbon payments (Khatri *et al.* 2018) and so-called scientific forest management practices (see Rutt *et al.* 2015). Over time there has been a gradual shift in the rhetoric from giving communities control over their forests to forest resources as they have recovered becoming increasingly commodified either for global programmes addressing climate change such as REDD+ (Reduced Emissions from Deforestation and Forest Degradation) (Khatri *et al.* 2018) or for revenue from timber or high value non-timber farm products (Paudel 2016).

From the start the authority that CFUGs have over their community forests has been circumscribed. The Forestry Department has a strong focus on the technical management of forest and communities have specified but limited rights and authority (Ojha 2008; Nightingale and Ojha 2013). When they are established and before they are entrusted with specified forest areas, CFUGs are required to prepare a constitution and submit a management plan for approval. Generally, the CFUG constitutions’ stated objectives are to ‘promote the scientific management of the forest as prescribed in the existing Act and Laws’ and ‘to fulfil forest product demand of the users by increasing the production of the forest’.^1^

Forest regulations (Ministry of Forests and Soil Conservation 1995) established what should be included in the management plan and these were backed up by Guidelines for the Inventory of Community Forests (Ministry of Forests and Soil Conservation 2000) ostensibly drawn up to assist CFUGs and District Forest Office field staff in assessing the condition of the forests, and that can best be described as classic forest inventories. The exercise of estimating annual increment and allowable cut has been largely fictional since the Forestry Department has limited capacity to implement the guidelines. There are reports that in preparing operational plans, foresters have admitted to reducing the stated height and diameter of standing trees in the inventory in order to reduce the estimate of allowable cut (Chettry *et al.* 2003:69) to keep in line with regulations on upper limits for growing stock volume (Baral et al., 2018). Management plans cover five to ten years and require a renewal process requiring technical skills that CFUGs are expected to organize and fund. However, many smaller CFUGs have limited resources to hire expert assistance, and consequently a significant number currently have no valid management plan, restricting any approved use of their community forest.

The plans and objectives of community forests have prioritised product and protection objectives and have not systematically addressed livelihood needs, designed effective mechanisms for benefit sharing, or accounted for the employment or income generation objectives of different social groups. Singularly absent has been any consideration for the potential contribution of forests to food security (Khatri *et al.* 2017). As secondary forest has recovered in the mid-hills of Nepal community forestry has been incorporated into the agenda of an emerging global policy to address climate change. This has reinforced the processes by which outputs from the secondary forest recovery are being appropriated and commodified and local governance of community forestry is increasingly subjected to external forces (Khatri 2018).

### Processes of Agrarian Change

There has been little debate about the drivers behind the increasing forest through secondary forest regeneration and the degree to which community forest management has been key to those changes (see Marquardt *et al.* 2016). There has also been limited attention given to the changing role of forests in the rural economy of the mid-hills. The literature has either focused entirely on the forest to the neglect of its interrelations with agriculture (Ojha *et al.* 2013; Pandey *et al.* 2014) or adopted a model of forest dependency that reflects the structure of the agrarian economy two decades ago (Adhikari *et al.* 2007; Dhakal *et al.* 20,011; Maraseni *et al.* 2014). While agriculture has stagnated, a global labour market has reduced the role of agriculture in rural livelihoods in the mid-hills but led to higher living standards for many. Changes in the agrarian economy have profound effects not only on the role of agriculture but also through various interrelated mechanisms shift demand for and use of forest products reflecting agroecology and the location specific nature of agrarian and forest change (Marquardt *et al.* 2016; Fox 2018; Khatri 2018).

While the forest boundary in the mid-hills of Nepal retreated in the past under agricultural pressure, there is now evidence of a reversal of this trend with forests extending into the agricultural landscape. This may be due to overall shrinking farm sizes – over one generation these have halved (Marquardt *et al.* 2016) - and declining livestock numbers. But there are also processes of input intensification linked to on-farm secondary forest regeneration (through intensive livestock management systems and greater use of farmyard manure) that have resulted in increasing farm productivity. At the same time a loss of labour from the rural economy through out-migration is occurring (Marquardt *et al.* 2020). Along with increased wildlife damage as a result of forest recovery, this is leading to more marginal land being abandoned for annual crop cultivation or converted to the management of trees and fodder.

### Social and Spatial Differentiation

Two mutually reinforcing processes have consolidated existing patterns of social differentiation based on caste and land ownership. The recovery of secondary forest has been in part due to restrictions put on grazing and access to other forest products that have contributed to a decline in livestock numbers, a shift to improved breeds, and an increase in on-farm tree cover for stall based livestock (Adhikari *et al.* 2007; Dhakal *et al.* 2011; Khatri *et al.* 2017). However, for the many landless households dependent on livestock extensive grazing in community forests is critical, as is forest access for collection of fuel wood and other NTFPs for subsistence or income.

The increasing commodification of community forests, whether through REDD+ payments (Khatri *et al.* 2018) or the promotion of timber and NTFPs (Paudel 2016) has primarily benefited village elites. There is often a lack of transparency within the CFUGs and the poor are often unaware of how decisions are made and who is making them (Khatri 2018). Where forest rents are high this may lead to a significant black economy and capture of forest rents (Iversen *et al.* 2006). The view that increased income alleviates poverty, reflected in so-called equity interventions promoted by external programmes such as REDD, may in fact encourage poor households to engage in the market through risky small livestock enterprises to their detriment (Nightingale 2017; Khatri *et al.* 2018). Degree of social differentiation, commodification, and their social consequences are village specific and reflect both agroecology and specific forest types as well as market penetration (Sugden *et al.* 2017).

In the mid-hill districts of Dolakha and Ramechhap, levels of outmigration are high. In some places, subsistence farming systems are gradually shifting to semi-commercial vegetable farming. The farming systems are connected to secondary forest through community forestry and trees on farmland as fodder for livestock. However, in the more resource rich districts in the Chure and terai areas, which consist of larger and denser forests with high value timber species such as Sal (*Shorea robusta*) and Asna (*Terminilia*), e.g., Sindhuli and Chitwan, there is more commercialization of forest management. The community forests are used not only to meet subsistence needs but the CFUGs also harvest timber for sale in the market. There is great interest from the forest bureaucracy in control of community forest activities (Ojha 2008; Nightingale and Ojha 2013). Forests in the Chure and *terai* are at greater risk of deforestation as residents draw on them for income, primarily from timber. However, in mountain districts, there is also some extraction of NTFPs at commercial scale.

## Peru

### Context

Peru has long been characterised by marked social distinctions that reflect its colonial history and the social divides among its coastal, Andean, and Amazonian territories where most of its forests are located. Although forest clearance started in the 1940s, it was not until the 1980s that road development, state sponsored colonization schemes, and market support led to a systematic expansion of the agricultural frontier (Coomes 1996; Alvarez and Naughton-Treves 2003; Chávez *et al.* 2014). This encouraged significant in-migration of people from the Andes escaping violence and land shortages (Menton and Cronkleton 2019) that in San Martín has led to a complex social mix of indigenous populations, a long-resident mestizos of mixed ancestry who arrived in the nineteenth century, and more recent Andean migrants who may now constitute nearly 50% of the region’s population (Paz y Esperanza 2015). Naturally regenerated secondary forests now comprise a significant portion of San Martín’s forest area.

Prior to the 1980s agriculture in San Martín was divided between small areas of semi-permanent cropping fields along the river and road corridors and swidden agriculture practiced by both mestizo and indigenous people in the forest areas (INEI 1997; Limachi *et al.* 2006). As agriculture has expanded from the valley and river plains into the adjoining forested hills indigenous people have retreated further into the forest. These shifts in the forest frontier have left land rights and forest access insecure and contested among the regional government, indigenous groups, Andean migrants, and expanding large scale oil palm plantations, and becoming a source of conflict.

### Governing the Forest

Traditional swidden farming systems (de Jong 2001; Padoch and Pinedo-Vasquez 2010; Coomes *et al.* 2017) in the San Martín region (Marquardt Arevalo 2008) are based on selective forest clearance, and bio-diverse fallowing practices generate areas of semi-managed secondary forest. However, these swidden systems have become increasingly differentiated with respect to farm size and fallow durations and quality (Marquardt *et al.* 2019). In more densely settled areas with limited forest area some farmers are now deliberately planting suitable tree species to shorten and improve fallow regeneration (Marquardt *et al.* 2013).

Historically the indigenous communities were semi-migratory living in the forest and drawing on its resources, including extensive territories for hunting. Although they are now primarily farmers, they culturally identify with the forest. The ‘Law of Native Communities and Agrarian Development in the Lower and Upper Rainforest’ of 1975 (Decree-Law 22,175) was enacted to comply with the ILO Convention No 169 to protect the territories and rights of the indigenous people, recognising them as *communidad nativa* (Crovetto 2007:11). Registration as a communidad nativa entitles villages to communal territory including forests, as well individual title for household properties. By 1997, however, few in San Martín had obtained legal recognition of their community status with forest territorial titles. A decade later driven by pressure on their lands an increasing number have achieved communidad nativa status, but without adjoining forest communal title.

Underlying the reluctance of the regional government to meet its legal obligations has been its programme to promote forest conservation and its demarcation of 70% of its territory as suitable for conservation apparently as a response to a target set by Peru’s National Forest Conservation Program for Climate Change (Gobierno Regional de San Martín 2016). There are various categories of forest conservation areas including regionally protected areas, national parks, areas for ecosystem conservation, recovery and conservation concessions, as well as native community recognition with communal forest title (Marquardt *et al.* 2019), all aimed at maintaining or restoring primary forest areas but excluding people and allowing restricted uses not including fallow based agricultural practices. These key conservation and ecosystem recovery zones include areas that the indigenous communities see as their ancestral territories and for which they have registered claims according to the law.

However, these designated protection and conservation zones remain largely on paper and the limited power of the regional government to create and enforce in practice them has left powerful actors able to subvert them to the detriment of indigenous communities. Kowler *et al.* (2016) have reported that oil-palm companies growing have been able to have land designated for conservation reclassified as agricultural. Some communities of migrants have been able to secure legal tenure even though they are in conservation areas. Increasing numbers of migrants have led to forest clearance and competition for land with indigenous and mestizo land holders.

Conservation zoning has become a threat to indigenous communities who have limited means to resist rather than an intervention that could support them in protecting their forest areas. They also face constant incursions particularly by migrant farmers into lands that they regard as their ancestral territories, but they have limited means to resist such settlement (Marquardt *et al.* 2019). The preferred path to secure their land rights is to obtain recognition as a native community and secure the land title to their community areas. But rather than granting indigenous communities legal title that would give them authority over their forest resources and land use, the regional authorities instead strongly prefers to award native community status in combination with forest conservation concessions for a fixed term and restricted use that do not allow any form of agricultural practice or guarantee any permanence of the use rights. This has generated debate within indigenous communities over whether they should continue to pursue efforts to obtain their legal rights.

One Kechwa-Lamas village filed a lawsuit in August 2017 to challenge the legality of the land titling procedures being pursued by the regional government in conservation areas. In response the regional government threatened to suspend all titling of Kecha-Lamas communities in the area in an effort to dissuade them from pursuing their legal rights but was finally forced to withdraw this threat under pressure from civil rights groups and the Kechwa-Lamas themselves.^2^ But there are also NGOs, often with a focus on conservation rather than indigenous livelihoods or human rights who are supportive of the government measures (Egerlid *et al.* 2016).

### Processes of Agrarian Change

There are multiple drivers underlying the competition for land and retreat of the forest boundary and several government policies have in effect supported the opening of the Amazon through the construction of roads, promotion of settlement, and support for the commercialisation of agriculture. Greater access to land and the development of a cash crop economy has provided a stimulus for the in-migration of the land poor and driven land use change (Menton and Cronkleton 2019). Government has actively promoted cultivation of perennial cash crops, notably coffee, cocoa, and oil-palm, leading to a significant expansion in their area and production (Gobierno Regional de San Martín 2017; INEI 2017). The area under coffee has tripled since 2000 and cocoa production has increased by a factor of 20. The assumption underlying the promotion of these perennial crops is that they would stop the expansion of the cultivation boundary, supplant the need for extensive cultivation including swidden agriculture and spare the forests. But this has not happened.

Instead in-migration supported by the availability of land and the expansion of coffee and cocoa production have contributed significantly to forest clearance. While the capital intensive oil-palm plantations are largely confined to the lowlands, there is evidence that they have often been established in primary forest areas, and any further expansion is likely push out existing smallholder settlements up into the forested hills of the Amazon (Kowler *et al.* 2016).

Indigenous communities and other smallholders have incorporated both coffee and cocoa cultivation into their cropping system (Marquardt *et al.* 2019, establishing permanent agroforestry fields alongside their fallowing systems. Where forest areas are more limited and fallowing systems of short duration, non-farm labour often in neighbouring urban economies has become a key source of income, but many households continue as part time farmers to meet their subsistence requirements and as a means to reinforce their claims to forest lands they consider their ancestral territory.

In sum the processes of agricultural intensification in San Martín have had perverse effects in relation to the forests. The establishment of perennial cash crops has led to a semi-permanent loss of both primary and secondary natural forest driven by market forces. The monocropping of these perennial species bears no comparison with the biodiverse secondary regeneration fallow systems of traditional smallholders (Marquardt *et al.* 2013), which arguably contribute to the maintenance of an Amazonian forest cover.

### Patterns of Social and Spatial Differentiation

There is limited data on and understanding of processes of agrarian differentiation and class structures with respect to land ownership within or between any of the indigenous, migrant, and mestizo communities. Indeed, there are major challenges to making such an analysis given the land extensive and transitory nature of swidden land use systems, the limited degree to which smallholders have secured legal land title and the rapid expansion of informal migrant settlements. However, since agricultural margins are still expanding in San Martín and land titles are not firmly established it is unlikely that major forms of class differentiation based on land holdings are emerging. However, there is clearer spatial patterning of differentiation with long-settled mestizo communities concentrated in the river valleys and plains primarily engaged in intensive annual crop production, indigenous communities primarily concentrated in the more forested uplands around the river valleys, and migrant communities pushing back the forest/agriculture boundary. Despite smallholder engagement in tree crop production, their primary focus is simple reproduction and meeting subsistence needs, suggesting that market forces have not yet fully penetrated San Martín’s rural landscape.

## Discussion

Central to our discussion of the Nepal and Peru case studies are the questions of what secondary forest is, where it should be, what it should be used for, by whom, and how. We address these issues from two perspectives. The first is a bureaucratic perspective which sets the administrative and particular knowledge framework regarding the forested rural landscape. The second is the perspective of the practitioners. Scott (1998) and Bhattacharya (2018), distinguished forest from agriculture, primary from secondary forest, and “proper” agricultural practices from “improper” ones. Swidden agriculture in Peru and tree management in farmers’ fields in Nepal are regarded as “improper.” But for the people on the ground what they actually do is simply common sense.

From the bureaucratic perspective the differences between forest and agriculture, and primary and secondary forests are established facts even though these categories are essentially a priori concepts that are imposed on the facts on the ground. For example, for taxation purposes in the Punjab the British colonial authorities created categories of space, agrarian structure, and land rights that were at variance with what was actually there (Bhattacharya 2018). This is similar to the San Martín regional government’s categories of land use created through remote sensing techniques, although it lacks the coercive power to enforce them, particularly in relation to migrants and agricultural capital, and its own policies actually undermines them. In Nepal, where there is a stronger bureaucracy, forests are delineated between community and government, although community forests are increasingly subject to scientific forest management or conservation standards that may disadvantage those without sufficient resources to implement them.

In Nepal the focus is on generating revenue from forests, including those under scientific forest management and those that can be used for payments for ecosystem services. Despite the diversity of secondary forests and the resources they provide to users, the interests of rural elites and the forest bureaucracy increasingly overlap to the detriment of local communities. In San Martín where the policy focus is on old growth forest, secondary forest is not treated “proper” even though it covers a greater area than primary forest (Kowler *et al.* 2016) In Nepal trees outside the forest are regarded to be on uncultivated land.

Specific political conditions generated the rise of community forest as a model in Nepal. The movement of labour out of the mid-hills altogether from 2000 onwards reduced pressures on the forests and favoured their recovery. If the overseas labour market disappears or is significantly reduced as is now happening with the effects of the Covid 19 pandemic could have significant consequences for the forests.

Different global environmental narratives contributed to the respective forest debates – Himalayan environmental degradation in the 1980s and global climate change and the role of the Amazon. The effects of global food regimes (McMichael 2013) and the promotion of tree crops for global markets that are a significant force now in San Martín were not present in Nepal in the 1990s. But in Peru these global processes have been exacerbated by deep social and structural inequalities of Peru, with marginalised populations in the both Andes and the Amazon. In the latter, however, global recognition of the rights of indigenous peoples have forced at least nominal concessions from the P,eruvian state in terms of recognition of their territorial rights. In San Martín the growth of the coca economy during the 1980s and 1990s was linked to forms of armed resistance to the state and transnational drug production and market networks and may have encouraged state support of in-migration and opening the region to global markets.

Migration is common to forest histories in both locations. In the Amazon there has been in-migration and seasonal migration for non-farm work. In Nepal demands of global labour markets have fuelled out-migration. In both cases households remain rural but increasingly draw subsistence income from non-farm sources. New forms of migration and the diversification of rural livelihoods render classic models of agrarian and forest transition redundant and point to the functional joining of forest and agriculture by rural households and the critical role of secondary forest in securing their livelihoods (Hecht 2014).

## Conclusion

Our findings from Nepal and Peru suggest current models of forests and agrarian transitions no longer reflect the facts on the ground, failing to recognise the diversity of secondary forest types and uses and what underlies their creation and maintenance within a landscape (Messier *et al.* 2015; Chazdon *et al.* 2016). This requires rethinking the categories of forest and agriculture and a greater focus on the integrity of mosaic landscapes and the people who live in them (Pokorny 2013). Trees in the transition zone between forestry and agriculture may offer greater opportunities for managing biodiversity and secure the future of both rural household livelihoods and the forests at the same time (Marquardt, 2008; van der Ploeg 2014). However, will only be through their ability to secure their legal rights that indigenous peoples and other smallholders will be able to secure access to forest resources in of the Peruvian Amazon, and rural households in Nepal can achieve equitable access the (see Thompson 1975).

## Footnotes

1 From the constitution of Dhuseri CFUG in Rupandehi, and common to all the CFUG constitutions that were examined at that time (ODG and Norms 2003).

2 http://www.forestpeoples.org/en/node/50283
